# Antibiotic susceptibility guided reuse of levofloxacin-based therapy in a penicillin-allergic patient for *Helicobacter pylori* infection: A case report

**DOI:** 10.1097/MD.0000000000024915

**Published:** 2021-03-12

**Authors:** Siya Kong, Han Chen, Keting Huang, Duochen Jin, Guoxin Zhang, Feng Ye

**Affiliations:** a Department of Gastroenterology, The First Affiliated Hospital of Nanjing Medical University; b First Clinical Medical College of Nanjing Medical University, Nanjing, PR China.

**Keywords:** *Helicobacter pylori*, levofloxacin, rescue therapy, susceptibility testing

## Abstract

**Rationale:**

Antibiotic resistance poses a challenge for *Helicobacter pylori* eradication treatment. Current guidelines strongly recommend avoiding repeated treatments with the same antibiotic to prevent the emergence of drug resistance. However, for penicillin-allergic patients with recurrent *H. pylori* eradication failures, avoiding repeated treatments with the same antibiotic severely limits the choice of treatment.

**Patient concerns:**

A 47-year-old woman with a penicillin allergy for whom 2 previous levofloxacin and bismuth-based therapies had failed.

**Diagnosis:**

*H. pylori* infection.

**Interventions:**

Agar dilution susceptibility testing and gene sequence analysis was performed to confirm levofloxacin susceptibility again. Therefore, we treated her with a 14-day regimen consisting of levofloxacin (500 mg once daily), furazolidone (100 mg twice daily), colloidal bismuth pectin (220 mg twice daily), and esomeprazole (20 mg twice daily).

**Outcomes:**

The patient was successfully treated with a third levofloxacin and bismuth-based regimen.

**Lessons:**

Antibiotics included in previous failed therapies need not be eliminated if no antibiotic resistance is found on antimicrobial susceptibility testing.

## 1 Introduction

Levofloxacin is a third-generation fluoroquinolone that is widely used in clinical practice. The rate of *Helicobacter pylori* resistance to levofloxacin has increased with the widespread use of fluoroquinolone antibiotics.^[1]^ A recent review of 66,142 *H. pylori* isolates from 65 countries demonstrated that the rate of primary and secondary resistance to levofloxacin was more than 15% in all World Health Organization regions.^[2]^ Therefore, consensus reports have suggested that for patients with previous antibiotic exposure, repeated treatment with the same antibiotic should be avoided in order to reduce resistance rates.^[1,3,4]^ However, a retrospective study of 293 patients with a history of 2 failed *H. pylori* treatments found that clarithromycin-based therapies were repeated in 178 (60.8%) patients, and levofloxacin-based therapies were repeated in 88 (30.0%) patients.^[5]^ Thus, in clinical practice, repeated treatment with the same antibiotic is common among patients with *H. pylori* treatment failure. However, in patients with several treatment failures, the fourth treatment option is worthy of careful consideration. For penicillin-allergic patients in particular, the choices for *H. pylori* eradication therapy are limited after the failure of several eradication regimens, due to the necessity of avoiding amoxicillin and the high prevalence of clarithromycin and metronidazole resistance (70.4% and 82.4%, respectively, in the United States).^[6]^ Although fluoroquinolone consumption has been associated with levofloxacin resistance, repeated treatment with levofloxacin still carries a 50% chance of drug sensitivity.^[7–10]^ Hence, the reuse of levofloxacin-based quadruple therapy as the fourth treatment is worthy of consideration, especially, for penicillin-allergic patients.

Here, we report a case of *H. pylori* infection that was successfully treated with levofloxacin and bismuth-based therapy after the failure of 2 previous levofloxacin and bismuth-based treatments. This case shows that antibiotics included in previous failed treatment regimens need not be eliminated if susceptibility is confirmed on antimicrobial susceptibility testing, and that it may be possible to reuse the same antibiotic in an appropriate rescue therapy regimen.

## 2 Case report

During a routine clinical visit, a 47-year-old woman presented with an approximately 2-year history of recurrent epigastric pain and dyspepsia. The patient reported that she had previously undergone eradication therapies for *H. pylori* infection 3 times, and that her ^13^C-urea breath test (^13^C-UBT) remained positive at the last follow-up. A review of her medical history showed that she was strongly positive for penicillin allergy on allergy testing. Therefore, she had initially been prescribed an eradication therapy regimen consisting of rabeprazole, bismuth, clarithromycin, and levofloxacin. After 6 months, she still tested positive for *H. pylori* on ^13^C-UBT. She then received another 2 rounds of rescue therapies for *H. pylori* eradication within the following year (Table 1), but all these treatments failed and produced no significant improvement in her symptoms.

**Table 1:** History of *Helicobacter pylori* eradication treatments in our patient.

Start date	Regimen, dose, and frequency	Duration
Feb 8, 2017	Rabeprazole (10 mg bid), colloidal bismuth pectin (220 mg bid), clarithromycin (500 mg bid), levofloxacin (500 mg qd)	14 days
July 5, 2017	Esomeprazole (20 mg bid), colloidal bismuth pectin (220 mg bid), clarithromycin (500 mg bid), bifidobacterium (420 mg bid)	14 days
Mar 8, 2018	Esomeprazole (20 mg bid), colloidal bismuth pectin (220 mg bid), clarithromycin (500 mg bid), levofloxacin (500 mg qd)	14 days

**Figure 1:**
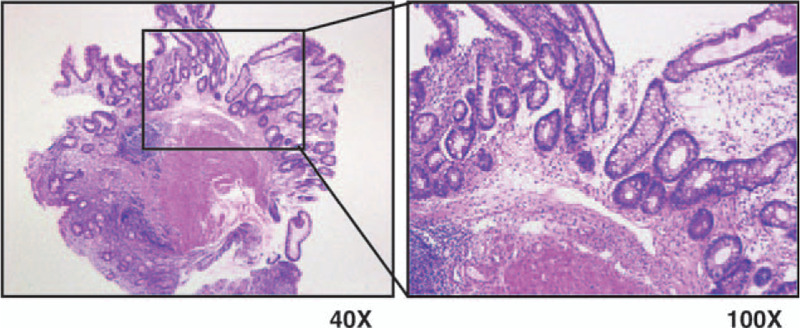
Histopathological examination of a gastric angle biopsy shows atrophic gastritis and intestinal metaplasia.

One month before the present visit, she underwent esophagogastroduodenoscopy with biopsy, and was histologically diagnosed with mild-to-moderate atrophic gastritis and intestinal metaplasia (Fig. 1). Additionally, a western blotting test was strongly positive for serum antibodies to the cytotoxin-associated gene A and vacuolating cytotoxin A.

**Figure 2:**
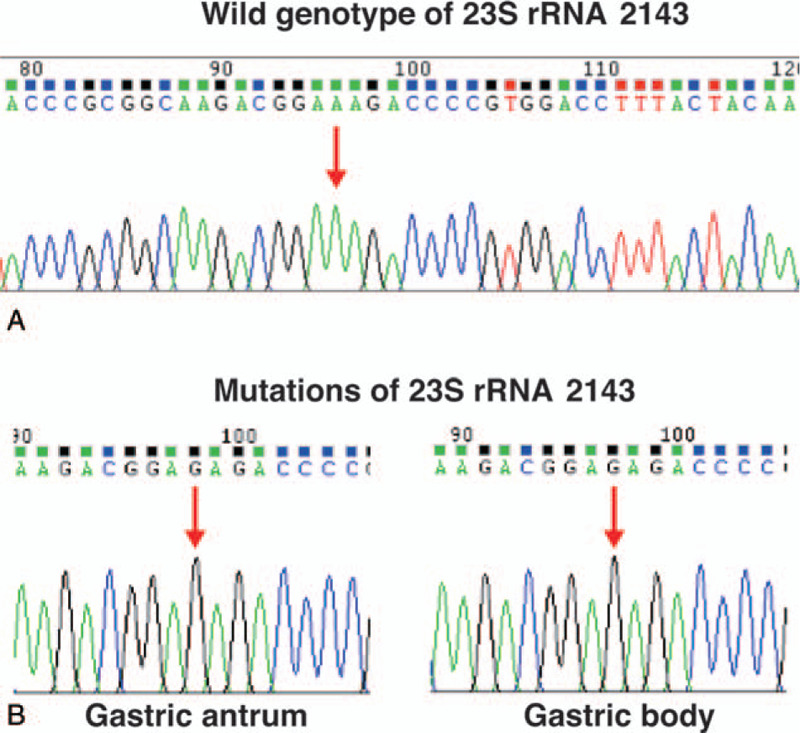
Sequencing results of the 2143 site of the *23S* rRNA gene.

**Figure 3:**
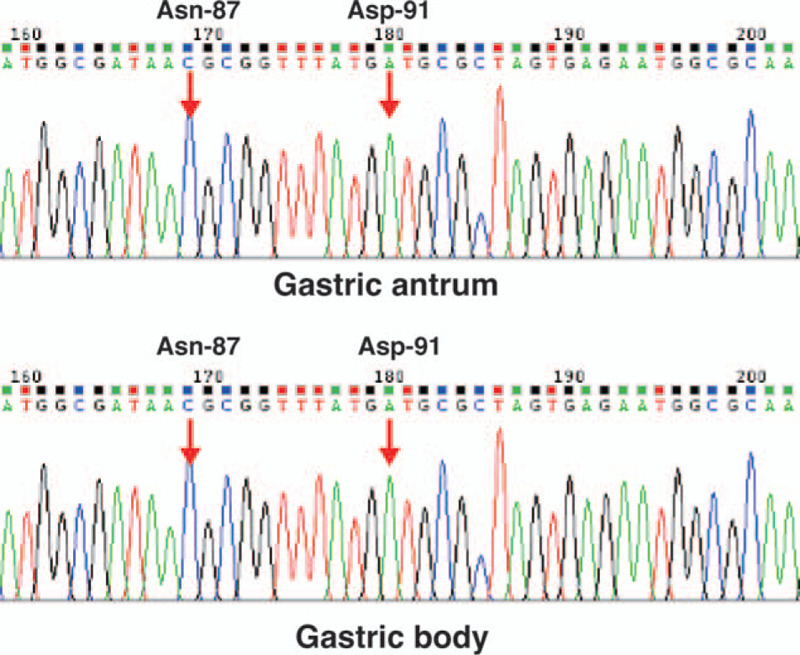
Sequencing results of Asn-87 and Asp-91 in the *gyrA* gene.

The patient agreed to undergo antibiotic susceptibility testing to minimize the risk of eradication failure. Written informed consent was obtained from the patient for all invasive treatments, and oral informed consent was obtained for the use of her medical data for scientific research, with her private information protected. The study protocol was reviewed and approved by the Ethics Committee of the First Affiliated Hospital of Nanjing Medical University. She subsequently underwent gastroduodenoscopy, and biopsy specimens were collected from the gastric antrum and body for *H. pylori* isolation. *H. pylori* culture-based antimicrobial sensitivity testing was conducted by the laboratory at Zhiyuan Inspection Medical Institute (Hangzhou, China), according to a previously described method.^[11]^ The results of agar dilution susceptibility testing showed susceptibility to amoxicillin, levofloxacin, and furazolidone and resistance to clarithromycin and metronidazole. The minimum inhibitory concentrations of the above drugs were as follows: amoxicillin, ≥2 μg/ml; levofloxacin, ≥2 μg/ml; furazolidone, ≥2 μg/ml; clarithromycin, ≥1 μg/ml; and metronidazole, ≥8 μg/ml. However, since the patient had twice received levofloxacin-containing regimens, gene sequence analysis was also performed to confirm levofloxacin susceptibility again. The molecular antimicrobial sensitivity test analyzed the sequence of the DNA gyrase A (*gyrA*) gene at Asn-87 and Asp-91 to determine levofloxacin sensitivity, and the sequence of the *23S* rRNA gene at site 2143 to determine clarithromycin sensitivity. In the case of the *23S* rRNA gene, we found the mutation genotype “G” instead of the wild-type genotype “A,” which indicated clarithromycin resistance (Fig. 2). In the case of the *gyrA* gene, the wild-type genotypes “C” at Asn-87 and “A” at Asp-91 were found, which indicated sensitivity to levofloxacin (Fig. 3). These results indicated that the patient's *H. pylori* strains were indeed sensitive to levofloxacin and resistant to clarithromycin, which was in accordance with the results of culture-based antimicrobial sensitivity testing. Therefore, we treated her with a 14-day regimen consisting of levofloxacin (500 mg once daily), furazolidone (100 mg twice daily), colloidal bismuth pectin (220 mg twice daily), and esomeprazole (20 mg twice daily). Two months after this therapy had ended, a 13C-UBT returned a result of 0.1 units; a result of <2.5 units was deemed to indicate successful treatment. Moreover, the symptoms of epigastric pain and dyspepsia had also significantly improved. Six months after ending the therapy, the 13C-UBT yielded a result of 0.7 units. Thus, finally, the eradication of *H. pylori* was successful.

## 3 Discussion

In this case, both the first and third treatment regimens contained clarithromycin and levofloxacin. Although both regimens had failed, antibiotic susceptibility testing demonstrated levofloxacin susceptibility and clarithromycin resistance. This indicated that clarithromycin resistance may be the most important determinant of eradication failure. Interestingly, the patient's *H. pylori* strains remained sensitive to levofloxacin even after 2 failed levofloxacin-containing regimens. This indicated that levofloxacin-sensitive *H. pylori* strains may not easily develop levofloxacin resistance. Indeed, levofloxacin was included in the patient's latest tailored therapy, which successfully eradicated the *H. pylori* infection.

Gram-negative bacteria, such as *H. pylori*, develop antibiotic resistance due to the transfer of antibiotic-resistance genes via mobile DNA elements such as plasmids, transposons, and integrons.^[12,13]^ In *H. pylori*, the main mechanism of fluoroquinolone resistance involves point mutations in the quinolone resistance-determining regions of the *gyrA* and *gyrB* genes,^[14]^ which change the effects of DNA gyrases (gyrA and gyrB) and topoisomerase IV.^[15]^ GyrA mutations at Asn-87 may confer a higher level of resistance to levofloxacin than mutations at Asn-91.^[16]^ In addition, gyrB mutations and several other gyrA mutations are not clinically important, and have a steady relationship with the gyrA 87 and gyrA 91 mutations.^[16,17]^ Our patient had no gyrA mutations at Asn-87 or Asp-91 after 3 eradication failures. A study of 28 patients with *H. pylori* infection and any prior fluoroquinolone use over the past 10 years found that 17 (61%) patients continued to have levofloxacin-sensitive *H. pylori* infection.^[18]^ Another study reported that suitable antibiotic exposure may not strongly contribute to the development of resistance.^[19]^ Considering these results and our present findings, we can confirm that if *H. pylori* isolates are found to be sensitive to levofloxacin, repeated treatment with levofloxacin may be justifiable, even for patients with a history of multiple treatment failures.

## 4 Conclusions

Although the American College of Gastroenterology guidelines recommend that in patients with *H. pylori* treatment failure, the same regimen should not be repeated,^[1]^ and the Toronto Consensus recommends against reusing levofloxacin for patients who have already failed to respond to a levofloxacin-containing regimen,^[4]^ this practice severely limits the choice of treatment for penicillin-allergic patients with recurrent *H. pylori* eradication failures. Antimicrobial sensitivity testing is a better guide for the selection of appropriate antibiotics for such patients. The present case provides additional evidence that the clinician can reuse antibiotics if susceptibility is confirmed, which is in accordance with the Maastricht V/Florence Consensus Report.^[3]^ Finally, the mechanisms underlying the development of levofloxacin resistance in *H. pylori* strains need to be further explored.

## Author contributions

**Data curation:** Feng Ye.

**Investigation:** Siya Kong, Han Chen, Duochen Jin, Feng Ye.

**Methodology:** Keting Huang.

**Supervision:** Keting Huang, Duochen Jin, Guoxin Zhang, Feng Ye.

**Writing – original draft:** Siya Kong.

**Writing – review & editing:** Han Chen, Guoxin Zhang, Feng Ye.
